# Intestinal Intraepithelial Lymphocyte Cytometric Pattern Is More Accurate than Subepithelial Deposits of Anti-Tissue Transglutaminase IgA for the Diagnosis of Celiac Disease in Lymphocytic Enteritis

**DOI:** 10.1371/journal.pone.0101249

**Published:** 2014-07-10

**Authors:** Fernando Fernández-Bañares, Anna Carrasco, Roger García-Puig, Mercè Rosinach, Clarisa González, Montserrat Alsina, Carme Loras, Antonio Salas, Josep M. Viver, Maria Esteve

**Affiliations:** 1 Department of Gastroenterology, Hospital Universitari Mutua Terrassa, University of Barcelona, Centro de Investigación Biomédica en Red de Enfermedades Hepáticas y Digestivas (CIBERehd), Terrassa (Barcelona), Spain; 2 Department of Pediatrics, Hospital Universitari Mutua Terrassa, University of Barcelona, Terrassa (Barcelona), Spain; 3 Department of Pathology, Hospital Universitari Mutua Terrassa, University of Barcelona, CIBERehd, Terrassa (Barcelona), Spain; 4 Department of Immunology, CATLAB, Viladecavalls (Barcelona), Spain; CNR, Italy

## Abstract

**Background & Aims:**

An increase in CD3+TCRγδ+ and a decrease in CD3− intraepithelial lymphocytes (IEL) is a characteristic flow cytometric pattern of celiac disease (CD) with atrophy. The aim was to evaluate the usefulness of both CD IEL cytometric pattern and anti-TG2 IgA subepithelial deposit analysis (CD IF pattern) for diagnosing lymphocytic enteritis due to CD.

**Methods:**

Two-hundred and five patients (144 females) who underwent duodenal biopsy for clinical suspicion of CD and positive celiac genetics were prospectively included. Fifty had villous atrophy, 70 lymphocytic enteritis, and 85 normal histology. Eight patients with non-celiac atrophy and 15 with lymphocytic enteritis secondary to *Helicobacter pylori* acted as control group. Duodenal biopsies were obtained to assess both CD IEL flow cytometric (complete or incomplete) and IF patterns.

**Results:**

Sensitivity of IF, and complete and incomplete cytometric patterns for CD diagnosis in patients with positive serology (Marsh 1+3) was 92%, 85 and 97% respectively, but only the complete cytometric pattern had 100% specificity. Twelve seropositive and 8 seronegative Marsh 1 patients had a CD diagnosis at inclusion or after gluten free-diet, respectively. CD cytometric pattern showed a better diagnostic performance than both IF pattern and serology for CD diagnosis in lymphocytic enteritis at baseline (95% *vs* 60% *vs* 60%, p = 0.039).

**Conclusions:**

Analysis of the IEL flow cytometric pattern is a fast, accurate method for identifying CD in the initial diagnostic biopsy of patients presenting with lymphocytic enteritis, even in seronegative patients, and seems to be better than anti-TG2 intestinal deposits.

## Introduction

An increase in intraepithelial lymphocyte (IEL) count per 100 enterocytes along villi is a cardinal diagnostic feature of celiac disease (CD), and it is the only abnormality found in Marsh type 1 lesion.[1], [2] However, it is not in itself sufficient for a definitive diagnosis of CD, as other pathologies may present in the same manner.[3]–[5] In this sense, other diagnostic approaches beyond conventional histology have been introduced for diagnosis of CD in the presence of a Marsh 1 lesion.[6], [7] The recent ESPGHAN guidelines for diagnosis of CD suggest that in these cases both a high γδ IEL count and the presence of IgA anti-tissue transglutaminase (anti-TG2) deposits in the mucosa increase the likelihood of a diagnosis of CD [8].

Assessment of the density of γδ IEL is in general performed with immunohistochemistry techniques in frozen biopsy samples.[2], [9], [10] Flow cytometry is a powerful analytical tool for the study of small intestinal immune cells and in particular the IEL, and it has been shown to be of value in the diagnosis of CD with atrophy, [11]–[13] and refractory CD.[14], [15] The advantages of flow cytometry are considerable compared to other user-dependent techniques, and results are obtained in a fast, sensitive, reproducible and objective semi-quantitative way just a few hours after taking the biopsy sample. It allows the analysis of a greater number of cells than does immunohistochemistry and yields a computerized record of the results. Using this technique, an IEL pattern typical of CD (CD IEL cytometric pattern) was defined, consisting of both an increase in γδ+ IEL and a dramatic decrease in CD3− IEL (reviewed by Leon F).[11] The γδ IEL increase is not totally specific to CD, since it has occasionally been found in other conditions such as cow’s milk intolerance, food allergy, cryptosporidiosis, giardiasis, Sjögren syndrome, and IgA deficiency.[11] However, the increase in γδ IEL in a minority of patients with these conditions tends to be mild and transient.[16] It has been stated that CD is the only disease in which γδ IEL are increased systematically, permanently, and intensely.[11], [17]–[19] The concomitant decrease in CD3-IEL provides increased specificity for the diagnosis of CD.[20] A description of this CD3-IEL population has been made, showing a CD3− CD7+CD103+CD45+ phenotype [12], [20], [21].

CD anti-TG2 specific auto-antibodies are produced at the local level in the small bowel mucosa. They can be found deposited below the epithelial basement membrane and around mucosal capillaries where they may be detected with immunofluorescence methods in a frozen biopsy sample.[7] This method seems to be very sensitive and specific in diagnosing CD, and the presence of these autoantibodies reinforces the diagnosis in borderline cases, mainly in seronegative CD [22]–[24].

Data about the usefulness of these new techniques in determining when lymphocytic enteritis is CD are scarce and have been limited to patients with positive serology.[6], [22], [25] However, it is well known that the sensitivity of celiac serology in Marsh type 1 lesion is low, [3], [8], [26], [27] and that when positive, a diagnosis of CD is generally definitive. To our knowledge, the reliability of IEL pattern analysis by flow cytometry in seronegative lymphocytic enteritis has not been investigated, and these are the challenging cases for CD diagnosis.

The aim of the present study was to evaluate prospectively the diagnostic accuracy of both CD IEL cytometric pattern analysis and anti-TG2 IgA subepithelial deposits for diagnosing CD in the form of both Marsh 3 and 1 lesions with positive serology. In addition, we assessed the usefulness of these parameters for diagnosing CD in patients with seronegative lymphocytic enteritis.

## Patients and Methods

### Patients and controls

Two-hundred and five consecutive patients (144 females; mean age, 29.3±1.3 years, range 1–79 years) who underwent small intestinal biopsy under clinical suspicion of CD and positive HLA genotyping (see below) were prospectively included in the period May 2010–December 2012. Clinical presentation is shown in Table 1. Fifty patients showed villous atrophy (Marsh classification type 3a, n = 5; and type 3b–c, n = 45) and received a diagnosis of CD on the basis of the rule of ‘4 of 5’ described by Catassi and Fasano [28]. Seventy patients showed architecturally normal small intestinal mucosa with an increase in IEL counts (lymphocytic enteritis, Marsh type 1 lesion): in 12 of them CD was suspected because of positive celiac serology, in 15 it was secondary to *Helicobacter pylori* infection, and in 43 the etiology of lymphocytic enteritis was unknown at inclusion. A clinical and histological response to eradication therapy was required to consider *Helicobacter pylori* as the cause of the enteritis. Eighty-five patients showed normal small intestinal mucosa; 8 of them had positive celiac serology.

**Table 1:** Clinical presentation of included patients.

*Clinical* *presentation*	*Atrophy* *(n = 50)*	*Lymphocytic* *enteritis (n = 70)*	*Normal histology* *(n = 85)*	*Overall* *(n = 205)*
Classical celiac disease*	12 (24%)	0 (0%)	1 (1.2%)	13 (6.3%)
Diarrhea	7 (14%)	21 (30%)	41 (48.2%)	69 (33.6%)
Dyspepsia	4 (8%)	18 (25.7%)	6 (7%)	28 (13.7%)
IBS symptoms	2 (4%)	12 (17.1%)	9 (10.6%)	23 (11.2%)
Iron-deficiency anemia	9 (18%)	11 (15.7%)	11 (13%)	31 (15.1%)
Growth failure	8 (16%)	0 (0%)	7 (8.2%)	15 (7.3%)
Abdominal pain	7 (14%)	6 (8.5%)	11 (13%)	24 (11.7%)
Asymptomatic – First degree relative	7 (14%)	6 (8.5%)	7 (8.2%)	20 (9.7%)
Organ-specific autoimmune disease	2 (4%)	0 (0%)	6 (7%)	8 (3.9%)
Systemic autoimmune disease	1 (2%)	3 (4.3%)	1 (1.2%)	5 (2.4%)
Increased level of liver enzymes	3 (6%)	3 (4.3%)	0 (0%)	6 (2.9%)
Microscopic colitis	1 (2%)	0 (0%)	1 (1.2%)	2 (0.9%)
Other	0 (0%)	0 (0%)	2 (2.4%)	2 (0.9%)

*Digestive symptoms and weight loss or growth failure.

The healthy control group consisted of 10 patients (8 women, 39.7±5.9 years, range 18–70 years) without CD (normal duodenal histology and negative celiac serology and HLA-DQ2/8). Eight additional patients with non-celiac villous atrophy were included as a disease control group. In these patients, atrophy was considered to be secondary to olmesartan use (4), collagenous sprue associated with collagenous colitis (1), ileal Crohn’s disease (1), or autoimmune disease-associated enteropathy (2). In all of them celiac serology was negative and there was no response to a gluten-free diet (GFD).

Patients with intake of non-steroidal anti-inflammatory drugs in the month previous to the endoscopy or with intestinal parasitic infection were excluded.

In all patients and controls sample biopsies from the 2^nd^–3^rd^ portions of the duodenum for both IEL flow cytometry and intestinal deposits of anti-TG2 IgA antibodies were obtained in the index endoscopy.

The protocol was approved by the Ethics Committee of the Hospital Universitari Mutua Terrassa and all participants (or their parents in case of less than 16 years old) provided informed consent.

### Flow cytometry

One single duodenal biopsy was obtained using a 2.8 mm biopsy forceps (Radial Jaw 4, Boston Scientific, USA), and immediately processed as previously described with minor modifications.[11], [20] Preparations of IEL suspensions were made by incubation with 1 mM EDTA, 1 mM DTT in HBSS for 90 minutes with continuous rotation at 12 rpm in a vertical shaker at room temperature. This procedure achieves total removal of villous epithelium and partial removal of crypt epithelium. The proper separation of epithelial compartment was confirmed by immunohistochemical analysis of the remaining tissue during the protocol validation. The obtained suspension, a mixture of IEL and epithelial cells, was washed once in fresh HBSS at 1500 rpm for 10 minutes, and IEL were immediately stained with previously titrated amounts of directly labeled antibodies for 15 minutes at room temperature. The antibodies used to define the different IEL subsets were anti-CD45-APC (clone 2D1), anti CD3-PerCP (clone SK7), anti CD103-FITC (clone Ber-ACT8) and anti-TCRγδ-PE (clone 11F2) (all from BD Biosciences, Franklin Lakes, NJ, USA). Intraepithelial origin of the IEL suspension was verified with CD103+ staining, and it was always ≥85%. Cells were immediately analyzed on a standard 4-color FACSCalibur instrument (BD Biosciences, Franklin Lakes, NJ, USA). Cell counts of the recovered cell number per biopsy were made with a hemocytometer and trypan blue exclusion. The average number of recovered IELs from one fresh biopsy was 353,258±13,841 (270,773±22,503 in patients with atrophy and 398,803±24,403 in those without atrophy).

**Figure 1:**
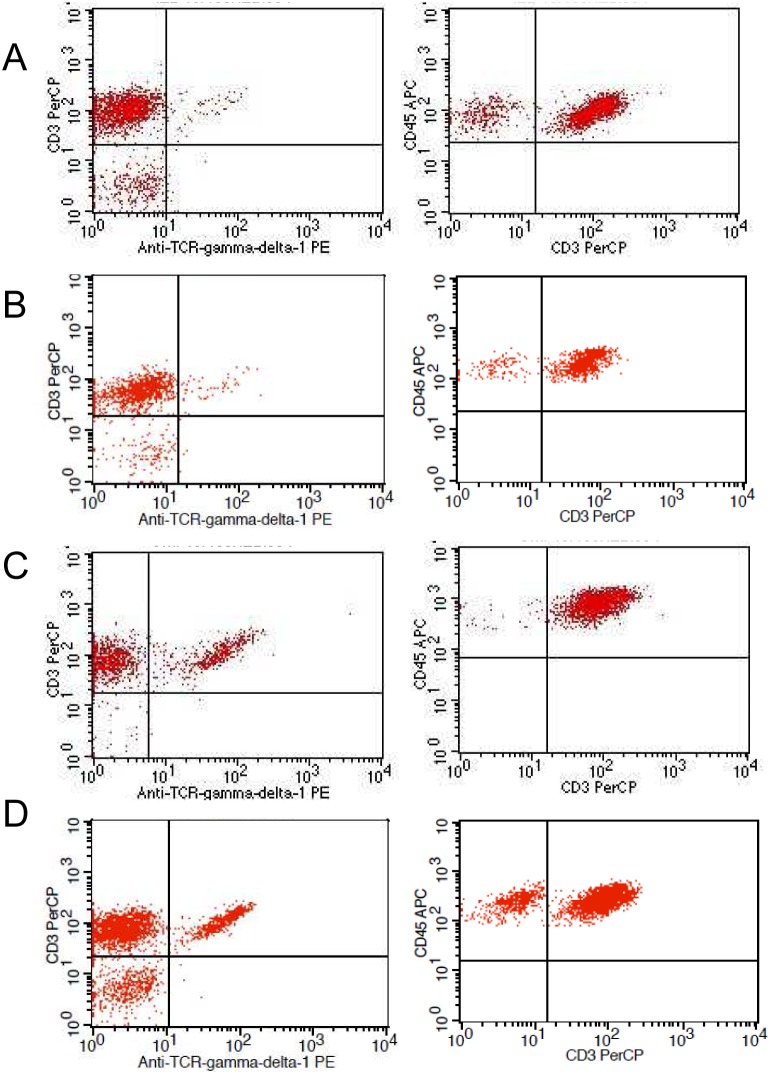
Representative flow cytometry dotplots of the 4 patterns. Left: TCRγδ+ T cells; Right: CD3− cells. Non-celiac patterns: A, Normal pattern; and B, Decrease of CD3− cells. Celiac patterns: C, Increase of TCRγδ+ T cells *plus* decrease of CD3− cells; and D, Increase of TCRγδ+ T cells only.

Results were obtained 3 to 4 hours after biopsy sampling, and were expressed as percentages over bright CD45 staining and low sideward scatter gate (Figure 1). The normal cut-off values for the IEL cytometric pattern in our laboratory are CD3+γδ+ IEL <8.5% (<mean+2SD) and CD3− IEL >10% (>10^th^ percentile). This cut-off was calculated in a sample of 65 non-celiac subjects. The intra-assay coefficient of variation is 5.5% (two replicates of each sample processed one immediately after the other), and the inter-sample coefficient of variation is 7.7% (two different samples from each patient obtained in the same procedure). Figure 1 shows the four cytometric patterns obtained. The complete CD cytometric pattern (Figure 1 C) was defined as TCRγδ≥8.5% and CD3–≤10%, whereas a selective increase of TCRγδ was considered as an incomplete CD cytometric pattern (Figure 1 D).

### Intestinal deposits of anti-TG2 IgA antibodies

**Figure 2:**
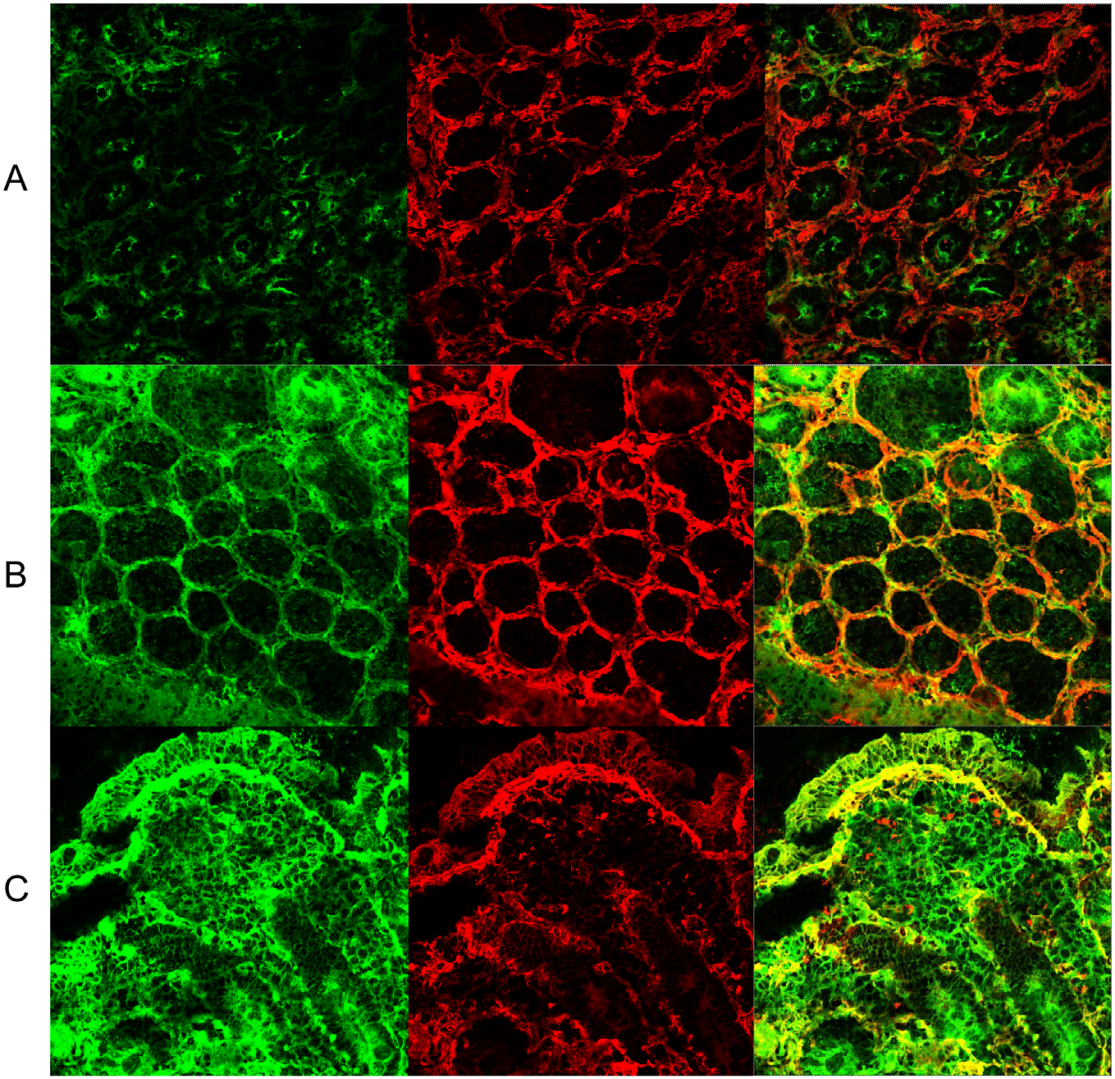
Intestinal deposits of anti-TG2 IgA. Immunofluorescence staining of IgA (green) and TG2 (red). Colocalization of IgA and TG2 is shown in orange-yellow. A: Normal histology patient with no deposits. B: Marsh 1 patient with mild staining around crypts. C: Marsh 3 patient with intense subepithelial deposits.

Biopsies were processed as previously described.[22]–[24] The evaluation of anti-TG2 IgA deposits was blindly performed on two occasions by two experienced observers, considering the pattern and the intensity of the staining as described.[24] In the non-concordant readings the highest intensity was considered. Figure 2 shows the immunofluorescence (IF) staining of intestinal deposits of anti-TG2 IgA antibodies in illustrative cases. A positive IF staining of anti-TG2 IgA deposits was considered as a CD IF pattern based on its high accuracy for CD diagnosis.[24] The degree of intra-observer and inter-observer concordance for positive deposits (low or high intensity) between the two anti-TG2 readings was substantial (Kappa = 0.75; 95% CI, 0.66 to 0.85; and Kappa = 0.79; 95% CI, 0.6–0.99, respectively).

### Celiac serology

Serum IgA-tissue transglutaminase antibody (anti-TG2) (or IgG anti-TG2 in IgA deficient patients) was analyzed in serum using a quantitative automated ELISA detection kit (Elia CelikeyTM, Phadia AB, Freiburg, Germany) with recombinant human TG2 as antigen. A value of anti-TG2<2 U/mL was established as the cut-off limit for normality.[29] Serum IgA anti-endomisial antibodies (EmA) were examined, as previously described, [30] in patients with anti-TG2 values ranging from 2 to 8 U/mL in order to confirm their positivity. Values above 30 U/mL were considered as high titers of anti-TG2. Total serum IgA was measured using rate nephelometry (BN II, Siemens Healthcare Diagnostics SL, Marburg, Germany).

### HLA genotyping

Genomic DNA from whole blood was purified using commercial Qiamp DNA Blood Mini kit (Qiagen, Düsseldorf, Germany). A commercial reverse hybridization kit for the detection of CD heterodimers HLA-DQ2 (A1*0501/*0505, B1*0201/*0202) and HLA-DQ8 (A1*0301, B1*0302) was used (GenID, GMBH, Strasburg, Germany). HLA-DQ2 haplotype is present in 24% of healthy controls and 90% of CD patients in our geographical area.[31] In the present study, positive celiac genetics indicates the presence of HLA-DQ2 (n = 145), HLA-DQ8 (n = 39), or HLA-DQ2 and DQ8 (n = 8). Some patients with only one allele of the HLA-DQ2 haplotype, either DQA1 (n = 3) or DQB1 (n = 10), were also included.

### Histological studies

Four endoscopic biopsies of the duodenum were processed using hematoxylin/eosin staining and CD3 immunophenotyping. Lymphocytic enteritis (Marsh type 1 lesion) was defined as 25 or more IEL per 100 epithelial nuclei along with normal villous architecture, as suggested in recent literature.[2], [3] IEL counts were performed as previously described.[3] *Helicobacter pylori* infection was investigated in gastric antral mucosal samples with standard histopathology assessment [32].

### ‘Gold standard’ for CD diagnosis

Suspicion of CD arose on the basis of a suggestive clinical picture and a positive genetic celiac study. The ‘gold standard’ in diagnosing a patient with CD was applied using the rule of ‘4 of 5’ described by Catassi and Fasano.[28] In this sense, villous atrophy or lymphocytic enteritis with positive serum anti-TG2 was considered as CD (titers between 2 and 8 U/mL were considered as positive only if confirmed by positive EmA).

### Statistical analysis

Results are expressed as mean (SEM) or as proportions (and their 95% confidence interval (CI) when appropriate). Diagnostic accuracy for CD diagnosis of intestinal IEL flow cytometry and anti-TG2 IgA intestinal deposits was calculated based on the following circumstances: 1) Presence of complete IEL cytometric CD pattern (TCRγδ≥8.5% and CD3−≤10%); 2) Presence of incomplete IEL cytometric CD pattern (TCRγδ≥8.5%); 3) Presence of positive subepithelial deposits of anti-TG2 (IF CD pattern); and 4) Presence of both TCRγδ≥8.5% and positive subepithelial deposits of anti-TG2 (combined incomplete cytometric/IF pattern). Sensitivity, specificity, positive predictive value (PPV), and negative predictive value (NPV) for CD diagnosis were computed on 2×2 tables. Patients fulfilling “4 of the 5” criteria for CD were considered as true positives (n = 60), whereas patients with non-celiac atrophy (n = 8) and patients with lymphocytic enteritis secondary to *Helicobacter pylori* (n = 15) were considered as true negatives.

McNemar test was used to assess differences in test sensitivity and specificity between the different tests. One-way analysis of variance was used for comparison of quantitative variables. As post-hoc tests to assess differences among groups, either the Bonferroni test for homogeneous variances or the Tamhane test for the non-homogeneous ones were used. Kappa coefficient was calculated as a measure of degree of concordance between the two readings of anti-TG2 IgA intestinal deposits. All statistics were generated using the SPSS for Windows statistical package (SPSS Inc., Chicago, IL, USA).

## Results

### Discrimination potential of intestinal intraepithelial lymphocyte flow cytometry and intestinal deposits of anti-TG2 IgA

**Figure 3:**
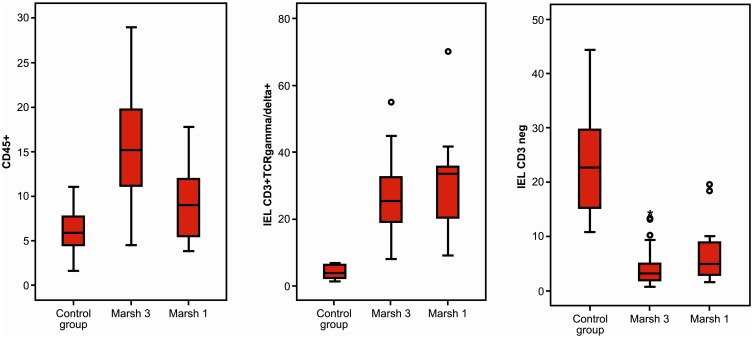
Comparison of IEL pattern between healthy controls, Marsh 3 patients and Marsh 1 patients with positive serology (serum anti-tTG2). In all cases p<0.001, representing the differences between Marsh 3 and Marsh 1 *vs.* control group.

The CD IEL cytometric patterns using the cut-offs described above are shown in Figure 1 (see patients and methods section). IEL cytometric pattern in Marsh 3 and Marsh 1 patients with positive serum anti-TG2 and controls is shown in Figure 3 and Table 2. CD45+ IEL percentage was not sensitive enough to separate CD Marsh 3 and Marsh 1 from healthy controls. By contrast, TCR γδ+ and CD3− cells allowed an excellent discrimination between patients and controls.

**Table 2:** Mean (±SEM) values of CD3+TCRγδ+ and CD3− intraepithelial lymphocytes in the different subgroups of patients with Marsh 3 and Marsh 1 type lesions as compared to healthy controls.

	*Control* *group* *(n = 10)*	*CD Marsh* *3 (n = 50)*	*CD Marsh* *1 Serology+* *(n = 12)*	*CD Marsh* *1 Serology−* *(n = 15)*	*Non-CD* *Marsh 1* *(n = 43)*	*p*
CD3+TCRγδ+ (%)	4.1±0.7	26.5±1.5^a^	31.4±5.1^a^	16.6±1.9^a^ ^b^	3.6±0.3	<0.001
CD3− (%)	23.7±3.5	4.1±0.5^c^	7.4±1.9^c^	7.5±1.4^c^	11.7±1.1^b^	<0.001

a p<0.05 *vs* Healthy controls and Non-CD Marsh 1;

b p<0.05 *vs* CD Marsh 3;

c p<0.05 *vs* Healthy controls.

There were no significant differences in the mean value of CD45+, TCR γδ+ and CD3− cells between children (<14 years) and adults (data not shown).

Regarding intestinal deposits of anti-TG2 (CD IF pattern) (Figure 2), non-concordant readings between the two evaluations performed occurred in 8 patients with normal histology, 9 patients with lymphocytic enteritis, and 5 patients with villous atrophy. Two of the 10 healthy controls (20%; CI, 5.6% to 50%) with negative HLA-DQ2/8 haplotypes showed low intensity positive deposits with agreement of the two observers.

### Diagnostic accuracy of CD IEL cytometric and IF pattern

Accuracy of the studied parameters for the diagnosis of CD in patients with positive serum anti-TG2 (Marsh 3 *plus* Marsh 1 lesions) is described in Table 3. We found that the highest sensitivity for CD diagnosis was achieved by the presence of an incomplete IEL cytometric pattern (97%), whereas both the complete CD IEL cytometric pattern and the combined incomplete/IF pattern had 100% specificity. Consequently, these criteria found to be highly specific for CD diagnosis were further applied to the diagnosis of patients with villous atrophy and lymphocytic enteritis.

**Table 3:** Accuracy of the parameters evaluated for the diagnosis of CD in patients with positive serum anti-TG2*.

	*Sensitivity %* *(95% CI)*	*Specificity %* *(95% CI)*	*PPV %* *(95% CI)*	*NPV %* *(95% CI)*
Complete FCP**	85 (73–92.5)	100 (82–100)	100 (91–100)	72 (53–86)
Incomplete FCP**	97 (87–99)^a^ **^,^** b	91 (70–98.5)	97 (87.5–99)	91 (70.5–98.5)
IF pattern**	92 (80–97)	87 (65–96.5)	95 (85–99)	80 (59–92)
Incomplete/IF pattern**	88 (77–95)	100 (82–100)	100 (91–100)	77 (57–89)

*Cases: 48 CD atrophy and 12 CD lymphocytic enteritis with positive serum anti-TG2; Controls: 8 non-CD atrophy and 15 lymphocytic enteritis secondary to *Helicobacter pylori* infection.

**Complete FCP: Complete CD IEL flow cytometric pattern (FCP): TCRγδ≥8.5% and CD3−≤10%; Incomplete FCP: Incomplete CD flow cytometric pattern (FCP): isolated TCRγδ increase (≥8.5%).

IF pattern: CD pattern of immunofluorescence showing intestinal deposits of anti-TG2 IgA; Incomplete/IF pattern: Incomplete FCP *plus* IF pattern.

a p = 0.06 *vs* Incomplete/IF pattern;

b p = 0.015 *vs* Complete FCP.

Two of the patients with non-celiac villous atrophy secondary to olmesartan use and unresponsive to a GFD had either an increase in TCRγδ+ IEL or anti-TG2 intestinal deposits; in addition, 3 of the 15 patients with lymphocytic enteritis secondary to *Helicobacter pylori* had one of the CD related parameters positive (2 anti-TG2 intestinal deposits and 1 increase in TCRγδ+ IEL).

### Patients with villous atrophy

**Figure 4:**
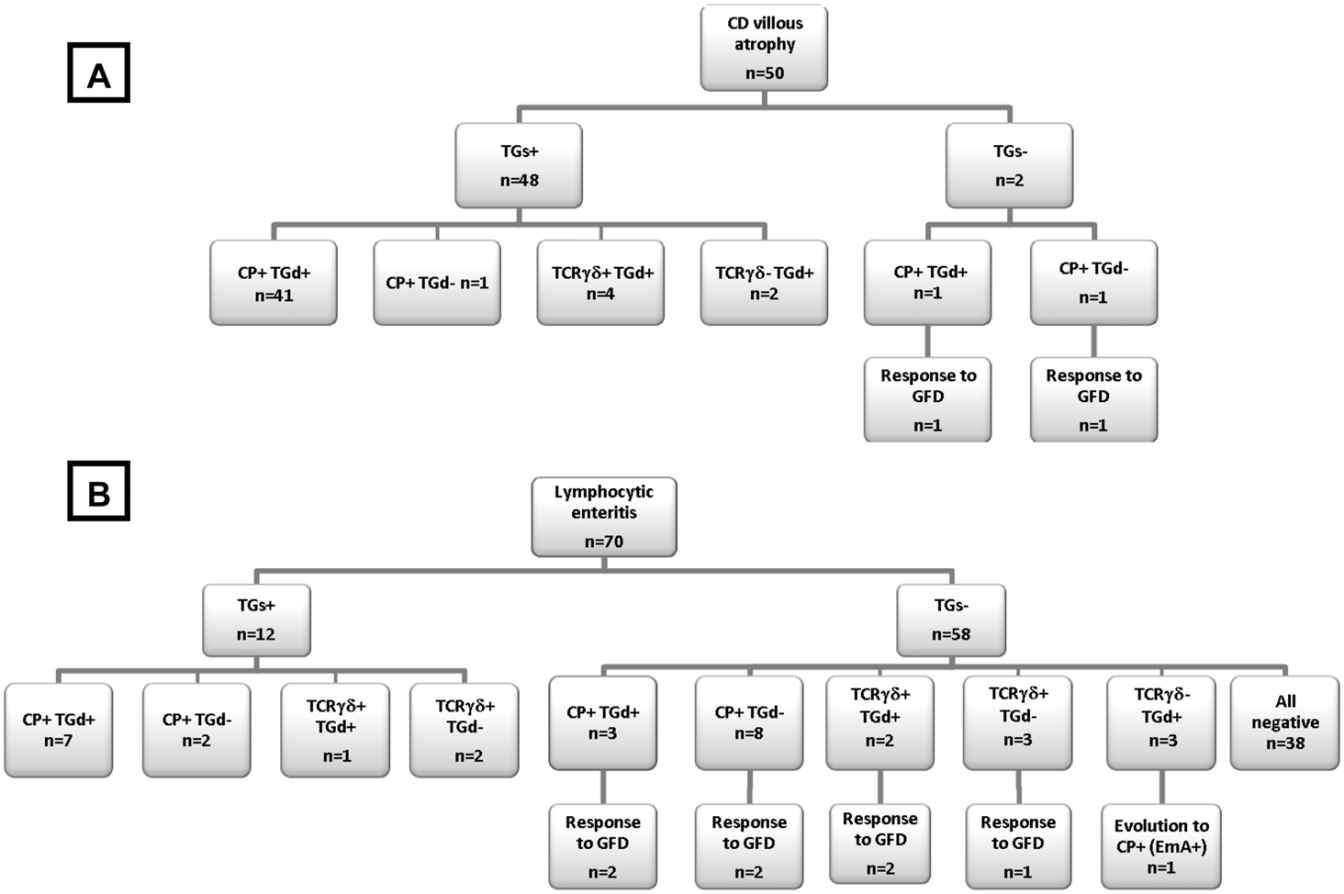
Flow diagnostic charts showing the presence of serum anti-TG2 (TGs), the complete CD IEL cytometric pattern (CP), an isolated increase of CD3+TCRγδ+ (incomplete CD cytometric pattern), and intestinal deposits of anti-TG2 IgA (TGd; CD IF pattern) in the studied population: A. CD villous atrophy; and B. Lymphocytic enteritis. Response to GFD is described when appropriate to fulfill the ‘gold standard’ (rule ‘4 of 5’).

Fifty of the 205 patients had villous atrophy. All of them fulfilled the ‘gold standard’ for CD diagnosis, 48 at baseline (i.e., before starting a GFD) and the remaining 2 after achieving a complete response to a GFD (flow chart in Figure 4A). Serum anti-TG2 IgA was positive in 48 of the 50 patients (44 at high titers and 4 at low titers). The CD cytometric pattern was present in 48 of the 50 patients (44 complete and 4 partial). In addition, a positive CD IF pattern was present in 48 of the 50 patients. Therefore, the simultaneous presence of the 3 analytical parameters, highly specific for CD diagnosis (serology, anti-TG2 intestinal deposits and complete IEL cytometric pattern), was found in 41 of the 50 CD patients with atrophy. In the remaining 9 patients at least one of these parameters was present showing their complementary value.

### Patients with lymphocytic enteritis

**Figure 5:**
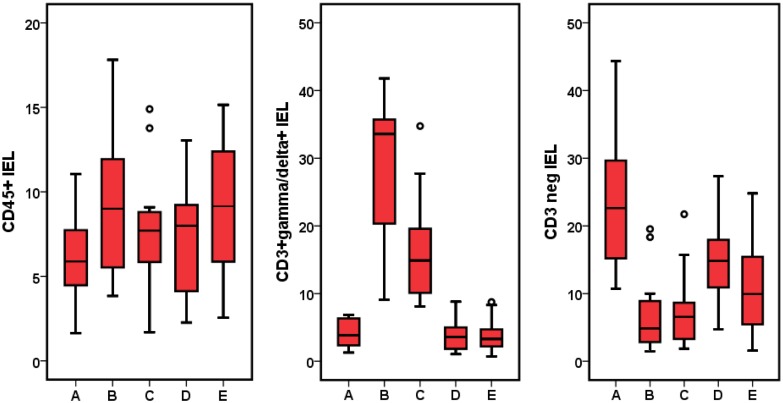
Comparison of CD IEL pattern between control group (A), seropositive CD Marsh 1 (B), seronegative CD Marsh 1 (C), lymphocytic enteritis secondary to *Helicobacter pylori* infection (D), and lymphocytic enteritis of unknown etiology (E). IEL CD3+γδ+: p<0.001, groups B and C *vs.* other groups; IEL CD3−: p<0.001, groups B and C *vs.* control group.

Seventy of the 205 patients had lymphocytic enteritis, 12 of them with positive serum anti-TG2 IgA. In Table 2 and Figure 5, results of IEL flow cytometry of patients with lymphocytic enteritis as compared to controls are provided. Seronegative CD Marsh 1 patients showed a significant increase in mean TCRγδ+ IEL as compared to healthy controls and non-CD Marsh 1, but the values were not as high as in CD Marsh 1 with positive serology. By contrast, the mean percentage of CD3− was identical in CD Marsh 1 irrespective of whether they had positive serology or not and was significantly lower than in healthy controls.

The routine immunochemistry anti-CD3+ IEL counting by the pathologist showed no significant differences in the different groups of patients with lymphocytic enteritis (Seropositive CD: 37.6±4.9%; Seronegative CD: 36.5±3.1%; *Helicobacter pylori* lymphocytic enteritis: 36.7±3.4%; other lymphocytic enteritis: 36.2±2%; p = 0.99).

#### Positive serum anti-TG2

All 12 patients fulfilled the ‘gold standard’ for CD (flow chart in Figure 4B; Table 4). Three of them had high titers of anti-TG2, 6 had low titers, and 2 had titers between 2 and 8 U/mL confirmed by EmA+; 1 patient with IgA deficiency had positive serum anti-TG2 IgG. The CD cytometric pattern was observed in 12 patients (9 complete and 3 incomplete pattern), and the CD IF pattern was observed in 8.

**Table 4:** Usefulness of both IEL flow cytometric pattern and IF pattern for the diagnosis of CD in patients with lymphocytic enteritis (Marsh 1 type lesion).

*Parameter*	*Serum* *anti-TG2+*	*Serum* *anti-TG2−*			*Overall*
	‘Gold standard’+n = 12	‘Gold standard’+n = 8	‘ProbableCD’ n = 7*	‘Non-celiac’n = 43	‘Gold standard’+n = 20
Complete FCP	9	4	7	0	13 (65%)**
Incomplete FCP	3	3	0	2	6 (30%)**
IF pattern	8	4+	1	3	12 (60%)
Incomplete/IF pattern	1	2	0	0	3 (15%)

FCP: Flow cytometric pattern.

*A GFD could not be started in 3 patients (1 not accepted, 2 non-symptomatic first degree relatives); Response to a GFD not evaluable in 4 patients (2 lost in follow-up, 2 with associated psychiatric disorders).

+ 1 patient with an isolated CD IF pattern at inclusion developed positive EmA and the complete CD cytometric pattern in a follow-up duodenal biopsy.

**Positive FCP (65%+30% = 95%) *vs* positive IF pattern (60%), p = 0.039 (McNemar Test).

#### Negative serum anti-TG2

Eight of the 58 patients with negative celiac serology fulfilled the ‘gold standard’ for CD since they had a good response to a GFD (flow chart in Figure 4B; Table 4). Seven of these patients had a CD cytometric and/or IF pattern; in the remaining patient serum EmA was detected and the IEL flow cytometry evolved to a complete CD cytometric pattern during follow-up.

In 7 additional patients CD was suspected since they presented the complete CD cytometric pattern and one of them also the CD IF pattern. However, the effect of a GFD could not be assessed in any of them (see Table 4). They were considered as ‘probable CD’ based on the high specificity of the complete CD cytometric pattern.

Forty-three patients were considered to have non-celiac lymphocytic enteritis. Two of them had a mild increase of TCRγδ+ IEL and three low-intensity positive anti-TG2 deposits.

Overall, the IEL flow cytometric pattern identified at baseline 19 of the 20 Marsh 1 patients who fulfilled the ‘gold standard’ for CD either at inclusion or after GFD, whereas both IF pattern and serology detected only 12 of them (95% *vs* 60% *vs* 60%; p = 0.039).

### Patients with normal histology

Eighty-five of the 205 patients had normal histology. Twelve of them had at least one of the three parameters found to be highly specific for CD. Eight had positive serology (7 with CD IEL cytometric pattern, 6 with CD IF pattern), 2 the complete CD IEL cytometric pattern and another 2 the combined incomplete IEL cytometric/IF pattern. These 12 patients were considered to have latent CD, with half of them being first degree relatives.

Twenty-two additional patients presented with only one celiac parameter: incomplete cytometric pattern in 17 and isolated IF pattern in 5. Nine of these 22 patients were first degree relatives of CD patients.

The cytometric values of the different subgroups of Marsh 0 patients as compared with healthy controls are provided in Table 5. As may be seen, there was a significant increase in mean TCRγδ+ IEL in latent CD as compared to healthy controls and non-CD Marsh 0, without significant changes in mean CD3− values. Values in non-CD Marsh 0 were very similar to those in healthy controls.

**Table 5:** Mean (±SEM) values of CD3+TCRγδ+ and CD3− intraepithelial lymphocytes in the different subgroups of patients with Marsh 0 type lesions as compared to the healthy control group.

	*Control* *group* *(n = 10)*		*Latent* *CD* *Marsh 0* *(n = 12)*	*TCRγδ+Marsh* *0 (n = 17)*	*IF* *pattern+Marsh* *0 (n = 5)*	*Non-CD* *Marsh 0* *(n = 51)*	*p*
CD3+TCRγδ+ (%)		4.1±0.7	19.0±2.7^a^	14.7±1.7^a^	4.8±0.9	4.5±0.3	<0.001
CD3− (%)		23.7±3.5	19.3±4.5	24.9±3.1	32.0±5.4	24.6±2.1	NS

a p<0.05 *vs* control group, IF pattern+Marsh 0, and Non-CD Marsh 0.

## Discussion

Differential diagnosis of lymphocytic enteritis is difficult, since multiple etiologies have been proposed for this condition [3]–[5]. Most patients have unspecific clinical symptoms, and a work-up to rule out CD is necessary. A low percentage of these patients have positive celiac serology, [3], [26], [27], [33] and others may progress to villous atrophy with positive serology after a gluten challenge.[5], [34] Both situations indicate that CD is a possibility to be considered. In most cases, however, the diagnosis of CD is obtained in seronegative patients with positive HLA-DQ2/DQ8 after showing a gluten dependence in both clinical symptoms and histology.[3] In spite of this, a diagnosis of CD frequently remains uncertain since the effect of a GFD on clinical symptoms is non-specific and lymphocytic enteritis may resolve spontaneously.[5] To further complicate this scenario, it has been recently reported that patients with non-celiac gluten sensitivity may have lymphocytic enteritis.[35] Therefore, tests to clearly differentiate celiac and non-celiac lymphocytic enteritis are needed.

In this setting, results of the present study suggest that the CD IEL cytometric pattern mainly disclosing an increase in TCRγδ+ IEL may be a useful tool for reinforcing the diagnosis of CD. The concomitant decrease in CD3− IEL as well as the presence of anti-TG2 intestinal deposits adds specificity for the CD diagnosis. Our results confirm the high diagnostic accuracy of both CD IEL cytometric and IF patterns in patients with atrophy and positive celiac serology, as suggested in the literature.[6], [11], [24], [36] These parameters were, in general, not necessary for CD diagnosis in patients with atrophy. However, the CD IEL cytometric pattern may be helpful in selected clinical situations such as cases with atrophy secondary to other etiologies. In this sense, in the present study 7 out of 8 seronegative patients with atrophy due to a variety of etiologies showed normal IEL pattern and negative deposits, and only 1 of these patients had an isolated increase in TCRγδ+ IEL without response to a GFD. Since atrophy cases in Western countries are generally due to CD, it is important to have complementary diagnostic tests to definitely rule out this diagnosis in doubtful cases with negative serology.

After the validation of the diagnostic usefulness of these parameters in patients with positive serology, we used them to categorize all included patients with lymphocytic enteritis as CD or not, on the basis of the 100% specificity for CD diagnosis of both the complete CD IEL cytometric pattern and the combined incomplete cytometric/IF pattern. This allowed us to observe that CD IEL cytometric pattern was more useful than both routine celiac serology and CD IF pattern for identifying CD in patients with lymphocytic enteritis. This methodology allowed us to establish the diagnose of CD in almost 40% of the patients with lymphocytic enteritis consecutively included in the present series, selected on the basis of clinical symptoms and positive celiac genetics. This is more than twice the number of patients diagnosed on the basis of serological results alone.

Results show that IEL subset assessment by flow cytometry was a reliable procedure with intra-assay and inter-sample coefficients of variation reflecting good performance on the test. Mean values of TCRγδ+ and CD3− cells observed in both the control group and patients with CD atrophy are very similar to what was previously described using the same methodology.[13], [20] The cut-off selected to define normal values was different from previous studies.[14], [37] In the first one the methodology was different since the entire biopsy specimen was homogenized and processed. In the second study, the IEL fraction was isolated as in the present study, but the cut-off used for TCRγδ+ was higher (<15%). The most likely explanations for this are differences in both the patients evaluated (Marsh 3 *vs* Marsh 1) and the selection criteria of the control group. In the present study, very strict criteria to select subjects for the control group were used to rule out latent celiac disease. Further, the mean TCRγδ+ value in CD Marsh 1 with negative serology was lower than in CD patients with positive serology, reinforcing the use of a lower cut-off to separate them from controls.

A normal IEL cytometric pattern was observed in control subjects and most of the patients with either non-celiac villous atrophy or *Helicobacter pylori-*associated lymphocytic enteritis, similar to what has been described in the literature.[11], [12], [20], [39] In fact, the presence of the complete CD cytometric pattern was associated with 100% specificity for CD diagnosis. Finally, sensitivity of TCRγδ+IEL increase for CD atrophy and CD lymphocytic enteritis was 97% and 95%, respectively. These figures are higher than those reported in the literature for the immunohistochemistry assessment of γδ+IEL to detect either CD atrophy or mild enteropathy CD in patients with positive serology. Those figures were 91–94% and 74–84%, respectively.[7] In addition, evaluation of γδ+IEL by immunohistochemistry in non-celiac controls is quite unspecific, yielding 18–23% positive results.[6], [36] In contrast, the present data and data from the literature suggest that the false-positive rate for γδ+IEL evaluation by flow cytometry is very low.[11], [12], [20], [38] Although further studies directly comparing the two techniques for evaluating γδ+IEL will be welcomed, the present data suggest that flow cytometry should be the preferred procedure to assess the γδ+IEL populations.

Anti-tTG2 IgA intestinal deposits fared worse than IEL cytometric pattern for detecting CD in patients with lymphocytic enteritis. It has to be taken into account that non-concordant results with the first reading negative and the second reading positive were considered as positive for the present study, which increased the positive result rate. Likewise, the reading of anti-tTG2 deposit plates was associated with some degree of subjectivity, and it lacked specificity. In this sense, the intra- and inter-observer concordance rates were substantial but not excellent, and 20% of control subjects without CD presented with positive deposits considered as false-positives. Previous studies showed excellent intra-observer and inter-observer concordance for the detection of anti-TG2 intestinal deposits, [6], [24] but non-celiac control subjects also presented positive mucosal deposits in 12 to 20% of cases.[6], [24], [36] The diagnostic yield observed in lymphocytic enteritis seems to be similar to or even worse than the diagnostic accuracy of the anti-tTG2 assay of the culture medium of biopsy specimens.[25], [26], [39] A recent study comparing the two techniques suggested that the measurement of antibodies secreted into culture supernatant is the best method for detecting intestinal anti-tTG2 antibodies [25].

A small subgroup of patients with positive celiac genetics and normal histology presented with highly specific CD parameters, half of them being first degree relatives of patients with CD, suggesting that they have latent CD, as has been previously described in the literature.[10], [11] There was another subgroup of these patients who presented with only one celiac parameter, either an increase in TCRγδ+ IEL or positive anti-TG2 deposits, many of them being also first degree relatives of patients with CD, leading to uncertain interpretation since a false positive result cannot be ruled out.

In conclusion, the analysis of IEL flow cytometric pattern is a fast, accurate method for identifying CD in the initial diagnostic biopsy of patients presenting with lymphocytic enteritis. In addition, it also allows detection of latent CD in subjects with normal histology. The diagnostic reliability of this test seems to be greater than that of the evaluation of anti-TG2 IgA intestinal deposits. Study highlights are described in Table 6. Further studies will be needed to confirm the accuracy of the CD cytometric pattern for the diagnosis of CD in patients with lymphocytic enteritis.

**Table 6:** Study highlights.

**What is current knowledge:**
• The differential diagnosis of lymphocytic enteritis may be difficult.
• Since celiac serology is often negative, a diagnosis suggestive of CD is obtained in patients with positive HLA-DQ2/8 after demonstrating the gluten dependence of both clinical symptoms and histology, which is a time-consuming and often disappointing diagnostic procedure.
• It has been suggested that both a high γδ IEL count with immunohistochemistry and the presence of IgA anti-tissue transglutaminase (anti-TG2) deposits in the mucosa may be useful to confirm the diagnosis of CD in patients with architecturally normal intestinal mucosa and positive serology for CD, but there are no data in seronegative CD patients.
**What is new here:**
**•** The analysis of IEL flow cytometric pattern is a fast, accurate method for identifying CD in the initial diagnostic biopsy of patients with lymphocytic enteritis.
**•** TCRγδ+ IEL quantification is significantly better than anti-TG2 IgA intestinal deposits for identifying CD in patients with lymphocytic enteritis, especially those with negative celiac serology.

## Supporting Information

Checklist S1 **STARD Checklist.**
(DOC)

File S1 **SPSS database.**
(SAV)
